# On Gout

**Published:** 1804-09-01

**Authors:** 


### *To the Editors of the Medical and Physical Journal.*

Gentlemen,

THE *liberal* discussion of a doubtful subject is the only probable mode of elucidating it; but how vague is the phrase, *liberal* discussion! Dr. Blegborough has given the public, in Number 65, an example of what he conceives to be a *liberal* discussion of the cooling treatment of gout, and promises renewed performances of similar *liberality*, if necessary. The observations which occur to me, as applicable to the subject, shall certainly not be *illiberal*, though they should chance to be inapposite or inconclusive.

Dr. B. sets off with an utter condemnation of a doctrine of gout, which indeed must be allowed to be as bold as novel; but it does not appear to me that either its boldness or its novelty involves its rejection without argument. It may, *as predicted*, possibly meet with *general opposition;* its originality invites it, but that general opposition will recoil on the opponents, if it should be founded on mere assertion, wholly unsupported by argument. It is not enough to aver that a doctrine is not tenable because it is at variance with popular opinion, it is requisite to impugn the principle upon which it rests, to brave its untenableness.

Not an opinion in physiology, however sanctioned, can be proof against unqualified attacks; nor is there one, however disputable, that ought to give way to so very sweeping an assault as that which Dr. B. has, in his own opinion, *liberally* opposed to the topical use of cold water in gout.

It is affirmed, that the gout is of local and not of constitutional origin; that the ligamentous and tendinous structure is necessary to its formal character, and that an inflammatory excitement of that particulars tructure is the painful source of the afflicting sympathies, which range more or less transiently throughout the system; it is also said that the proximate cause of all this grievance is excessive temperature, generated and evolved in the part affected by the agency of remote causes, according to the native laws by which heat is generated in every fibre of the animal economy.* All this, it must be confessed, is new, not unplausible, and has a very generalizing tendency respecting inflammatory affection; though it does not, as Dr. B. insinuates, reject the idea of specific inflammation; on the contrary, it founds the characteristic features of gout on the disease of a *peculiar fabric;* in that respect therefore it is specific, or *sui generis,* as Dr. B. will have it; but its specific nature is structural only, and not *material,* like syphilitic and cancerous virus, as he would wish to establish. A vast difference subsists between the simple excitement resulting from excessive heat, and that which arises from the virulent combination of animalized fluids: in the one case common irritation only prevails; in the other, complicated and durable disease; yet both are specific, but not similarly so, nor equally morbid. So much for a brief abstract and defence of the theory which espouses the

---

* See Remarks on Gout, by Dr. Kinglake, in the Medical and Physical Journal, No. LXI. pp. 241—246.

the practice of keeping inflamed gout in a state of reduced temperature.

What does the practice itself report? It affirms, (and that not only on the personal experience of the author of this view of the nature and treatment of the disease, but also on that of numerous correspondents) that it has in various instances proved rapidly and safely curative. Not one case has been offered to the public, excepting that by "A Constant Reader," of its deleterious effects; and that *solitary one*, unfortunately for the general disposition to oppose the practice, has been admitted to be wholly unfounded.

Dr. B. certainly refines on the gross humoral notion of gout, when he talks of "nervous irritability" and "obstruction;" but does he illustrate the nature and cause of the disease by these elaborate and inexplicable suppositions? He rejects the notion of morbid matter, and substitutes that of *specific laxity;* but how this specific laxity contrives to travel about the system, and occasionally, as if by intelligent predilection, to occupy particular stations, the Doctor does not so precisely specify; nor does he explain how this *itinerant laxity* suddenly braces itself up, and marches on to a new position. Surely, all this is *obscurum per obscurius;* nor is it conceivable how the Doctor, imagining gouty diathesis to be *specific laxity*, could, with common consistency, have stumbled on steam for its remedy. An application, unquestionably, as well adapted to soften rigidity, as to increase laxity.

The Doctor also speaks of "accumulated susceptibility," of "the left handed" erysipelatous inflammation, and of old people being insusceptible of excessive heat.

This is theoretic declamation only, and in no respect practically applicable. An unexcited heat does not amass excitability; on the contrary, it verges on inaction and consequent death, for want of excitement. Erysipelatous inflammation accurately denotes, by its greater or less violence, the exciting state of the constitution in which it occurs; but it is invariably an inflammatory affection characterized by excessive heat, and most directly curable by its due diminution; nothing sinister or "left-handed," as he, Dr. B. terms it, arises from this description of treatment; its salutary effect is uniform, definite, and in general complete. The thermometer, and what is more accurate, the natural sense of feeling, will discover a degree of heat in advanced age fully equal to what could occur in the utmost vigour of youth in similar morbid affections; fections; nay, the excess is often so great in old subjects, as actually to decompose organic structure by its combustive force.

Dr. B. is wedded to a preconceived opinion of the nature of gout, which it may be feared will unconsciously disqualify him for *liberally* discussing a doctrine that so directly militates against that which he has espoused. But the public, on the present occasion, is the jury; the merit of the refrigerant treatment of gout will be arraigned at that tribunal, by the evidence of facts *alone*, which will preclude all adverse surmises, and leave the issue to the unsophisticated claims of impartial justice.

Adhuc sub judice lis est.

*St. James's Street, July* 12, 1804. CANDIDUS.

